# Co-delivery of carbonic anhydrase IX inhibitor and doxorubicin as a promising approach to address hypoxia-induced chemoresistance

**DOI:** 10.1080/10717544.2022.2092234

**Published:** 2022-07-17

**Authors:** Muhammad Umair Amin, Sajid Ali, Muhammad Yasir Ali, Dominik C. Fuhrmann, Imran Tariq, Benjamin S. Seitz, Eduard Preis, Jana Brüßler, Bernhard Brüne, Udo Bakowsky

**Affiliations:** a Department of Pharmaceutics and Biopharmaceutics, University of Marburg, Marburg, Germany; b Department of Chemistry, Angström Laboratory, Uppsala University, Uppsala, Sweden; c Faculty of Pharmaceutical Sciences, GC University Faisalabad, Faisalabad, Pakistan; d Institute of Biochemistry I, Faculty of Medicine, Goethe-University Frankfurt, Frankfurt, Germany; e Punjab University College of Pharmacy, University of Punjab, Lahore, Pakistan

**Keywords:** Hypoxia, CA-IX enzyme inhibition, co-delivery, liposomes, mesoporous silica nanoparticles

## Abstract

Hypoxia, an oxygen-deprived condition of the tumor, is one of the major reasons for resistance to chemotherapy. Carbonic anhydrases are generally involved in pH homeostasis in normal conditions, but in solid tumors having a strong relation with hypoxia, the carbonic anhydrase IX (CA-IX) enzyme is overexpressed and results in an extracellular acidic environment. For most weakly basic anticancer drugs, including doxorubicin (Dox), the ionization in an acidic environment limits their cellular uptake, and consequently, the tumor exposure to the drug at sub-therapeutic concentration comes out as chemoresistance. Herein, a combined drug delivery system of liposomes and mesoporous silica nanoparticles (MSNPs) was developed for the co-delivery of the CA-IX enzyme inhibitor and Dox in hypoxic condition. The unique structure of MSNPs with higher surface area was utilized for higher drug loading and sustained release of Dox. Additionally, the biocompatible nature of liposomal coating as a second loading site for the CA-IX enzyme inhibitor has provided gatekeeping effects at pore opening to avoid premature drug release. Lipid coated MSNPs as a co-delivery system for Dox and the CA-IX inhibitor have synergistic cytotoxic effects against MDA-MB 231 breast cancer cells in hypoxic conditions. These findings assure the potential of this drug delivery system to overcome hypoxia-related chemoresistance.

## Introduction

**Scheme 1.**
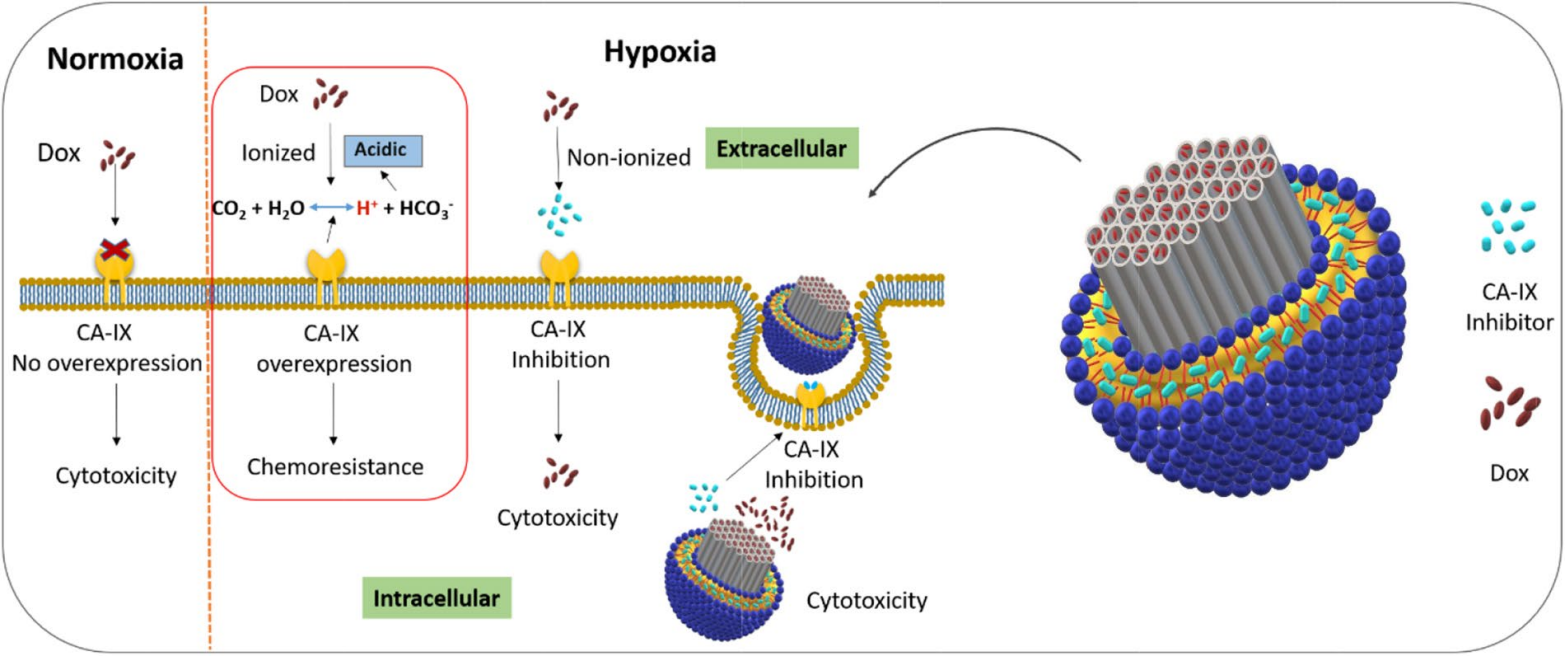
Representation of lipid coated mesoporous silica nanoparticle for co-delivery of carbonic anhydrase IX inhibitor and doxorubicin in hypoxia to overcome chemoresistance.

Hypoxia, the common feature of solid tumors, is a negative factor that is strongly associated with enhanced malignancy because of resistance to chemotherapy and ionizing radiation (Pettersen et al., 2015; Li et al., 2018). There is a strong evidence that hypoxia-inducible factor (HIF) and alteration in tumor metabolism linked with carbonic anhydrase enzyme IX and XII can play a vital role in tumor metastasis and progression (McDonald et al., 2016). Carbonic anhydrase IX (CA-IX) enzyme, a membrane protein of cancer cells, is rarely expressed in normal tissues but overexpressed under hypoxic conditions (Mahon et al., 2015; Pastorek & Pastorekova, 2015). For homeostasis CA-IX transports intracellular CO_2_ to the extracellular environment after converting it to carbonic acid and proton (Akocak & Ilies, 2014; Pastorekova & Gillies, 2019), as shown in Scheme 1. However, overexpression of CA-IX results in an extracellular acidic environment which has already been reported for different cell lines in hypoxic conditions. CA-IX, an endogenous marker for various tumors, has been under investigation to overcome the challenges of chemical marker administration and invasive procedures before a biopsy (Gieling et al., 2013; van Kuijk et al., 2016; Hu et al., 2020). Different studies revealed that hypoxic conditions of solid tumors trigger various gene expressions, including the CA-IX enzyme. The overexpression of CA-IX in the hypoxic tumor is associated with tumor aggression, poor prognosis, and radio- and chemotherapy resistance (Akocak & Ilies, 2014; Muz et al., 2015; Shabana et al., 2018). Most chemotherapeutics are weak electrolytes, and their uptake to the cells usually is by passive diffusion, but an extracellular acidic environment results in the ionization of such drugs. The permeability of the plasma membrane to ionized substances is very low because of ion trapping, which consequently results in lesser cellular uptake of the drugs (Lucien et al., 2017). According to this ion trapping model, weakly basic drugs such as doxorubicin are highly accumulated in the extracellular acidic environment, resulting in lower toxicity because of limited drug uptake (Mahoney et al., 2003).

Recent developments in silica-based nanomaterials have emerged as a very active domain for controlled drug delivery and other biological applications. Mesoporous silica nanoparticles (MSNPs) are desirable candidates because of their unique structure (Tang et al., 2012). MSNPs offer a larger surface area for higher drug entrapment and have dual-functional surfaces, including internal porous and external particle surfaces. These surfaces can easily be functionalized and conjugated to targeting ligands to achieve controlled release and target specificity, respectively (Li et al., 2013; Valtchev & Tosheva, 2013; Jin et al., 2017; Zhou et al., 2018). Both the hydrophilic and hydrophobic nature of MSNPs make them suitable candidates for the entrapment of a wide range of drugs. Polymeric nanomaterials have a limitation of drug leakage because of higher biodegradability, but MSNPs, offering mechanical strength and nontoxic behavior, have become promising drug delivery systems (Vivero-Escoto et al., 2010; Tang et al., 2012). These features of MSNPs assure high loading capacity for therapeutic agents and controlled release of drugs. MSNPs, other than polymeric nanomaterials that interact with organic solvents, are stable in both aqueous and organic solvents (Lu et al., 2007).

As drug delivery carriers, liposomes are other promising nanomaterials and have remained under consideration because of improved therapeutic efficacy with minimum side effects, rapid degradation, and enhanced drug absorption (Filipczak et al., 2020). The CA-IX enzyme inhibitor used in this study is a hydrophobic benzene sulfonamide and can reside in the lipid layer of liposomes. The outstanding features of liposomes compared to other nanocarriers resolve many issues related to drug delivery and diagnosis (Lamichhane et al., 2018; Tariq et al., 2019). Despite the liposomes’ salient features, safe delivery of highly toxic drugs during circulation remains a significant concern (Pattni et al., 2015; Ali et al., 2020). Various studies have shown that combining two different nanocarriers to form a new drug delivery system has produced promising results (Dragicevic & Maibach, 2018). The approach of coating liposomes on MSNPs enhances the stability of liposomes. On the one hand, a higher amount of drug can be loaded into MSNPs because of the larger surface area, and on the other hand, the lipid layer can also be used to entrap another drug (Liu et al., 2016). Lipid coated MSNPs can enhance biocompatibility and improve cellular uptake to the tumor cells (Cauda et al., 2010; Pattni et al., 2015). Lipid coated MSNPs have proved to be an ideal therapeutic delivery system, which synergistically enhances drug loading, stability, and controlled release of higher concentrations of multiple drugs to the target site. Similar synergetic effects of lipid coated MSNPs have already been reported compared to non-lipid coated MSNPs (Cauda et al., 2010; Wang et al., 2013).

Different approaches have been adopted for some anticancer drugs to alter the solid tumors’ internal and external pH and enhance their therapeutic effects. Extracellular pH enhancement was found with improved cytotoxic effects of weakly basic drugs. Carbonic anhydrase inhibition has shown synergistic effects in combination with chemotherapeutic agents (Corbet & Feron, 2017; Kolosenko et al., 2017; Supuran, 2018). Scheme 1 illustrates an advanced drug delivery carrier with carbonic anhydrase enzyme inhibitor-loaded in the lipid layer and doxorubicin in the mesoporous silica nanoparticles for co-delivery.

## Materials and methods

### Materials

Lipids including 1,2-dipalmitoyl-sn-glycero-3-phosphocholine (DPPC) and 1,2-dioleoyl-3-trimethylammonium-propane (DOTAP) were kind gifts from Lipoid GmbH (Germany). Carbonic anhydrase IX enzyme inhibitor, cholesterol, and 3-(4,5-dimethylthiazol-2-yl)-2-5 diphenyltetrazolium bromide (MTT) dye were purchased from Sigma Aldrich Chemie (Germany). Cetyltrimethylammoniumbromide (CTAB, >99%), dimethylsulfoxide (DMSO, ≥99.5%) and sodium hydroxide (NaOH, >99.5%) were purchased from Carl Roth GmbH & Co. KG (Germany). Tetraethylorthosilicate (TEOS, ≥99%) and doxorubicin hydrochloride (Dox, >95%) were purchased from Merck KGaA (Germany) and Fluorochem Ltd (UK), respectively. MDA-MB 231 cells used in experiments were obtained from ATTC® (USA). Dulbecco’s Modified Eagle’s Medium (DMEM) was purchased from Biochrom GmbH (Germany). Fetal calf serum was purchased from PAA Laboratories GmbH (Germany). Carbonic anhydrase IX monoclonal antibody was purchased from Santa Cruz Biothechnology, Inc (Germany). Tubulin and protein assay kits were purchased from Sigma Aldrich Chemie (Germany) and Bio-Rad (Germany), respectively.

### Fabrication of MSNPs

MSNPs were prepared by an adapted two-step sol-gel method catalyzed in a basic environment (Amin et al., 2021). Briefly, 0.67 mmol of CTAB were dissolved in 48 mL of purified water, and the solution was heated to 80 °C for 2 h with stirring at 350 rpm. When CTAB was dissolved entirely, and the milky solution became clear, 350 µL of 2 M sodium hydroxide was added. Later 2.2 mmol of TEOS was added dropwise with continuous stirring and heated overnight. The milky solution was then centrifuged at 16000 *g* for 20 min, and particles were collected as a pellet. For surfactant removal, particles were dispersed in acidic ethanol solution and refluxed at 80 °C for 6 h. After centrifugation, MSNPs were washed twice with ethanol and finally dispersed in purified water for lyophilization. Dried MSNPs were stored at −20 °C and used for further experiments.

### Preparation of CA-IX inhibitor liposomes (Lip_c_)

CA-IX inhibitor-loaded liposomes were prepared by using the thin-film hydration method (Mahmoud et al., 2018). Lipid solutions of DPPC, cholesterol, and DOTAP, dissolved in chloroform:methanol (2:1), were mixed in a molar mass ratio of 85:12:3. The organic solvents were evaporated at reduced pressure with a rotary evaporator at 45 °C to form a lipid film. Hydration of lipid film was done, and 1 mL PBS buffer (pH 7.4) was added, followed by sonication at 45 °C for 15 min. Then, liposomes were extruded with a 100 nm pore size polycarbonate filter at the transition temperature. A similar method was adopted to prepare CA-IX inhibitor-loaded liposomes with the addition of 20 nmol of CA-IX inhibitor dissolved in methanol with an organic lipid solution. The CA-IX inhibitor used in this study is a benzene sulfonamide and its binding affinity to different isozymes along its structure (Figure S1) has been given in Supplemental material. The final concentration of CA-IX inhibitor in liposomes was 20 µM and the final concentration of lipids was 10 mg/mL.

**Figure 1.**
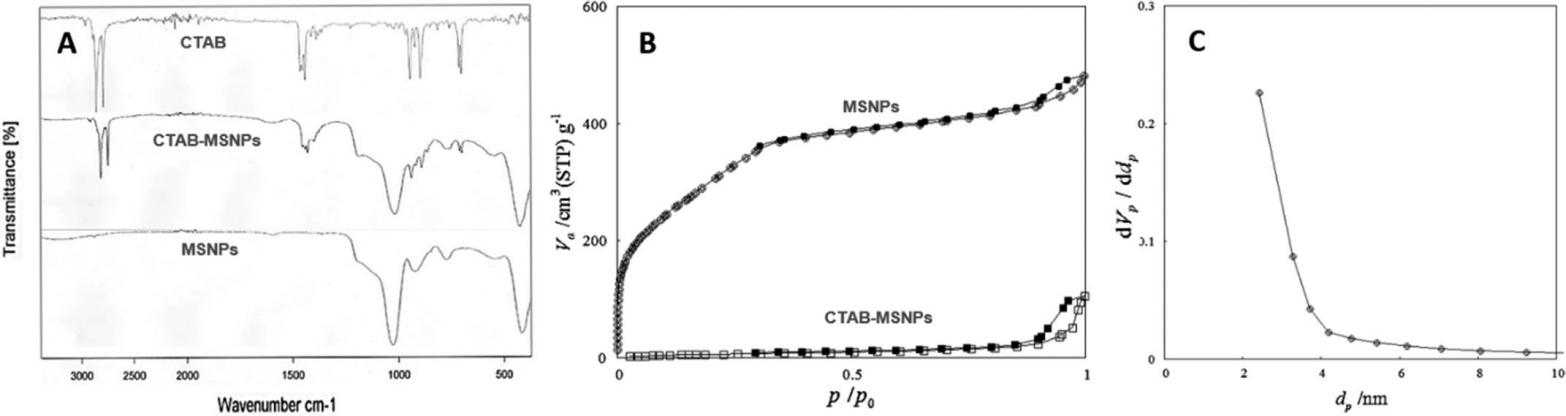
Image representing (a) FTIR spectrums of CTAB, CTAB-MSNPs and MSNPs, (b) MSNPs Nitrogen Adsorption-desorption isotherms before and after washing (c) BJH graph showing the pore size.

### Drug loading to MSNPs

For MSNPs drug loading, Dox, a weakly basic drug, was used as a model drug. 2.5 mg of dried MSNPs and 3 mg of Dox were dispersed in 6 mL purified water and sonicated for uniform dispersion. The mixture was stirred overnight at 350 rpm under light sealed conditions at room temperature. The Dox-loaded MSNPs were centrifuged at 16000 *g* for 30 min, and the supernatant was collected. The pellet was redispersed in water to remove the extra drug, and the process was repeated twice. The supernatant was then measured with a UV/Vis spectrophotometer for unentrapped Dox at 495 nm, from which the amount of entrapped drug was calculated (Yuan et al., 2011). The entrapment efficacy was calculated with the following formula, and drug-loaded MSNPs were lyophilized and stored at −20 °C.

$$\%\mathrm{Entrapment}=\frac{\mathrm{Drug}(\mathrm{entrapped})}{\mathrm{Drug}(\mathrm{total})}\times 100$$

The loading capacity of MSNPs was also calculated with the following formula.

$$\%\mathrm{Loading}\mathrm{capacity}=\frac{\mathrm{Drug}(\mathrm{entrapped})}{\mathrm{Weight}of\mathrm{MSNPs}}\times 100$$

### Preparation of Lip_c_-Dox-MSNPs

For Lip_c_ coating of MSNPs, a previously optimized molar mass ratio of MSNPs:liposomes (1:0.7) was used (Amin et al., 2021). After adding a specific amount of liposomes to dried Dox-MSNPs by pipetting, the mixture was sonicated for 10 min. The same procedure was adopted for the coating of Lip_c_ to Dox-MSNPs, and samples were stored at 4 °C. The CA-IX inhibitor concentration was kept above the minimum inhibitory concentration (Ki = 0.9 nM) despite any dilution step.

### Physicochemical characterizations

The physicochemical properties, including size distribution and zeta potential, were evaluated by Dynamic Light Scattering (DLS) and Laser Doppler Velocimetry (LDV), respectively, with the Zetasizer Nano ZS (Malvern Panalytic, Germany). Samples were diluted 1:100 with 1% phosphate buffer saline (PBS) and analyzed with folded capillary cuvette at 25 °C. Measurements involved automatic angle adjustment and laser attenuation by the device. Each measurement consisted of 15 runs for the hydrodynamic diameter, while zeta potential measurements included 15–100 runs. The data is represented as an average of three independent measurements.

FTIR spectroscopy of different samples was performed with Bruker-ALPHA ATR-FTIR (Bruker, Germany). Small amounts of samples were placed on ATR crystal to obtain spectrums of pure surfactant, MSNPs with and without surfactant, whereby surfactant removal could be assessed

The surface area with pore size and pore volume were characterized with the nitrogen adsorption-desorption technique using BELSorp mini II (BEL GmbH Europe, Germany). The sample was pretreated at 120 °C before measurement. The Brunauer Emmett Teller (BET) method was adopted for the surface area measurements and Barrett-Joyner-Halenda (BJH) method for evaluating pore size and pore volume. Nitrogen and Helium gases were used for purging and adsorption, respectively.

The morphological studies of MSNPs were performed by Transmission Electron Microscopy (TEM) at 300 kV (TEM, JEOL 3010), and the porous structure was visualized. MSNPs were dispersed in deionized water at 1 mg/mL, and 6 µL of the dispersion was placed on a holey copper grid and dried overnight to remove the liquid. Later on, it was coated with a thin layer of carbon because of the nonconductive behavior of silica. After placing the grid in the vacuum chamber, images were captured from different fields.

For the characterization of MSNPs’ size and lipid coating to MSNPs, AFM Nanowizard 3 Nanoscience (JPK Instruments) was used for visualization. Samples were diluted with water at a concentration of 1:100, and a small amount of diluted sample was placed on a silica wafer, fixed on a glass slide by double tape. After the sample settled, the extra liquid was removed with lint-free wipes and dried in the air. Cantilever tips HQ:NSC14/AL_BS & HQ:NSC16/AL_BS were used to measure the atomic force microscopy. Amplitude signals of the cantilever in trace direction and height signals in retrace direction were used to obtain the images.

## *In vitro* drug release

*In vitro* drug release studies were performed, and the drug release behavior from nanocarriers was evaluated. Different drug-loaded formulations corresponding to 3 mg of Dox were diluted to 15 mL of PBS buffers of different pH (5.5 and 7.4). The dispersions were incubated with stirring at 100 rpm at 37 °C in an orbital shaker (KS4000 IC, IKA Werke, Staufen, Germany). The samples were withdrawn at different time intervals and centrifuged at 16000 *g* for 30 min. The supernatant was collected, and the assessment of Dox release was done by measuring the absorbance at 482 nm by UV/Vis spectrophotometer (Ali et al., 2019). The pellet was redispersed in PBS (pH 5.5 or 7.4) and added to shaking dispersion to maintain the initial volume. Similarly, the release of CA-IX inhibitor was evaluated with PBS buffer of pH (5.5 & 7.4). As CA-IX inhibitor is lipophilic so 0.1% inert surfactant (Tween 80) was used to solubilize it. Samples were taken at different time intervals and after centrifugation, supernatant was measured at 275 nm with UV-visible spectrophotometer. The results are presented as mean of three independent measurements.

## Serum stability studies

The stability of nanoformulations was evaluated in the simulated physiological conditions by using different mediums with different concentrations of FCS. Briefly, 200 µl of formulations (Lip_c_ and Lip_c_-Dox-MSNPs) were diluted in 1 ml of DMEM with 10% FCS and 60% FCS at volume ratio of 5. In addition, the formulations diluted with PBS pH 7.4 at same volume ratio were used as control. The mixtures were incubated in a shaking incubator with a speed of 100 rpm at 37 °C (KS4000 IC, IKA Werke, Staufen, Germany). The samples were taken at different time intervals and diluted 1:20 with PBS pH 7.4 buffer. Subsequently formulations were measured for their size, PDI and zeta potential with Nano ZS Zetasizer. The results were obtained for three independent formulations and represented as mean ± SD (Abd Ellah & Abouelmagd, 2017; Ali et al., 2021).

### Cell culture experiments

*In vitro* cell culture experiments were performed with breast carcinoma cell line MDA-MB 231. Cells were grown in DMEM medium containing 1% non-essential amino acids (NEA) and 10% fetal calf serum (FCS) in T-75 culture flasks. Cells were maintained in 7% CO_2_ under a humidified atmosphere at 37 °C. The medium was changed on alternate days, and cells were sub-cultured upon 80% confluency. For the oxygen-deprived environment, cells were transferred to hypoxic conditions and maintained in SciTive Hypoxic Workstation (Baker Ruskinn, Leads, UK) with 1% O_2_, 5% CO_2,_ and residual N_2_.

#### Extracellular acidification test

As the overexpression of the CA-IX enzyme in hypoxia results in an extracellular acidic environment, an extracellular pH evaluation was performed to validate hypoxia. The effects of drug delivery systems on pH were investigated before and after exposure of the cells to formulations under hypoxia or normoxia. Cells were seeded in 6-well plates at the density of 25 × 10^4^ cells/cm^2^, divided into two groups for hypoxic and normoxic conditions, and incubated for 24 h. Later on, cells were incubated with different formulations diluted with 1.5 mL of medium for 4 h and 24 h. The pH of each well was measured before and after treatment. The final Dox and CA-IX inhibitor concentrations in each sample were 100 µg/mL and 360 nM, respectively.

#### Immunoblotting

The overexpression of the CA-IX enzyme in hypoxic conditions was analyzed with western blot. Cells were seeded in 6-well plates with a density of 25 × 10^4^ cells/cm^2^ and maintained under hypoxic or normoxic conditions for 24 h. Afterward, the medium was removed, and cells were washed with PBS buffer. Later on, cells were harvested, and a pellet was obtained, subsequently lysed with lysis buffer containing 4% SDS, 100 mL Tris/HCl, and 150 mM NaCl at pH 7.4, and stored at −20 °C. After determining total protein concentration with a protein assay kit (Bio-Rad, Munich, Germany), western blot was performed to evaluate CA-IX and HIF1α expression. Briefly, 50 µg of protein was loaded to 10% SDS gel and blotted with Trans-Blot Turbo blotting system (Bio-Rad). The membrane was blocked with 5% milk in TBS-T for tubulin (sigma). The CA-IX (H-11) monoclonal antibody was used as the primary antibody against the CA-IX enzyme (Rami et al., 2013; Fuhrmann et al., 2018). Similar procedure was adopted after treatment with different formulations to assess the inhibition of CA-IX with 24 h incubation time.

## *In vitro* cytotoxicity (MTT assay)

Cell viability assay was performed in hypoxia and normoxia to evaluate the *in vitro* cytotoxic effects of different formulations. Briefly, MDA-MB 231 cells were seeded in 96-well plates with a cell density of 1 × 10^4^ cells/cm^2^ and incubated overnight under normoxic or hypoxic conditions. The next day, after medium aspiration, formulations dissolved in 1.5 mL of the medium were added, and cells were incubated for 4 h and 24 h. Subsequently, the old medium was replaced with fresh medium, and cells were incubated overnight. On the following day, the medium was removed, MTT dissolved in the medium was added, and the well plates were incubated for 4 h. Finally, formazan crystals were dissolved in DMSO, and the absorbance was measured with a plate reader (FLUOstar, BMG Labtech GmbH, Germany) at 570 nm to evaluate the viability of the cells. The untreated cells in the medium were considered as blank with 100% cell viability (Ali et al., 2020). The amount of Dox used in each sample was 100 µg/mL The molarity of the CA-IX inhibitor was 360 nM, which is higher than the minimum effective concentration of the CA-IX inhibitor (Ki = 0.9 nM).

### Cellular uptake studies

The fluorescence-based quantitative analysis of cellular uptake of Dox was done with flow cytometry. Cells were seeded with a final density of 5 × 10^6^ cells/cm^2^ in 6-well plates for hypoxia or normoxia. Plates were incubated overnight, and after 80% confluency, the old medium was replaced with formulations corresponding to 50 µg/mL Dox. After 4 h incubation, cells were washed with PBS buffer and detached with trypsin EDTA. Subsequently, cells were centrifuged, and the pellet was resuspended in 1 M HEPES buffer (pH 7.9). Samples were subjected to FACSCanto II (BD, USA) for flow cytometric analysis, and detailed results were obtained by FACSDiva software. The measurements were based on the excitation and emission of Dox at 488 nm and 560 nm, respectively. The histogram of each sample was generated by using 10,000 gated events. Cells with a normal medium were considered as control, and finally, the mean fluorescence intensity of Dox is represented as the average of three independent measurements.

The intracellular localization of Dox was also evaluated with Confocal Laser Scanning Microscopy (CLSM) images. Cells were seeded in a 6-well plate with coverslips at a final density of 4 × 10^5^ cells/cm^2^. For comparison, plates were exposed to normoxic or hypoxic conditions. After 24 h incubation medium was replaced with PBS buffer for washing, and finally, cells were treated with free Dox, Lip-Dox-MSNPs, and Lip**_c_**-Dox-MSNPs containing 25 µg/mL Dox for 4 h. Later on, cells were thoroughly washed with PBS buffer to remove adsorbed substances. Following the fixation of cells by 300 µL of 4% paraformaldehyde with 10 min incubation, cells were washed with PBS buffer. Cells were incubated with 4′, 6-diamidino-2-phenylindole (DAPI) for 25 min for nuclei staining. Cells were further washed with PBS buffer, and a coverslip was placed on a glass slide with the addition of FluoSave, an aqueous mounting medium. The samples were observed under CLSM (Zeiss, LSM 700) with an excitation/emission at 359/457 nm for DAPI and 480/590 nm for Dox.

### Hemolysis assay

The hemolytic effect of different formulations on erythrocytes was determined by hemolysis assay. After the donor’s consent, the whole blood sample was collected in an EDTA tube. The erythrocytes were isolated by centrifugation in pellet form, which was washed thrice with PBS buffer (pH 7.4). After diluting 1:50 with PBS, erythrocytes were transferred to a V-bottom microtiter plate. Subsequently, they were incubated with different formulations corresponding to 50 µg/mL Dox for 1 h at 37 °C in an orbital shaker KS4000 (IC (IKA Werke). The plate was then centrifuged, and the supernatants were measured for absorbance at 540 nm using a microplate reader (FLUOStar Optima). PBS buffer (pH 7.4) and 1% Triton X-100 were used as the negative and positive control, respectively.

### Activated partial thromboplastin time (aPTT)

aPTT test was performed, and the effect of formulations on the coagulation time was determined. A previously described protocol was adopted using Coatron M1 coagulation analyzer (TECO, Neufahrn, Germany) (Alawak et al., 2020). After prior consent of the donor, fresh blood was withdrawn in a 3.8% sodium citrate tube. The plasma fraction was separated by centrifugation at 1500 *g* for 10 min. Later on, 25 µL of plasma was mixed with 25 µL of different formulations, followed by the addition of 25 µL of aPTT reagent. Finally, coagulation was activated by adding an equal volume of pre-warmed 25 mM calcium chloride solution. The coagulation time was determined spectrophotometrically, and the results are presented as an average of three individual values.

## Statistical analysis

All experiments were performed in triplicate unless otherwise stated and results are represented as mean ± SD. One-way analysis of variance (ANOVA) with post hoc test (Tukey’s multiple comparison) was applied by using Dox as control for Coagulation time and Lip_c_-Dox-MSNPs for Hemolysis assay. Two-way ANOVA with Tukey’s test (multiple comparison between the means) was performed for extracellular pH change, MTT assay and Dox (MFI) cellular uptake assay. Graph Pad Prism 6 was used for the statistical analysis and the rejection of null hypothesis was considered with a significance levels of *p* < 0.05.

## Results and discussion

### MSNPs synthesis and characterization

The fabrication of MSNPs was done by slightly modifying the method reported by Ma et al., where TEOS was used instead of TMOS because of the slower hydrolysis rate of the ethyl group compared to the methyl group. CTAB was transformed to micellar form with a cationic charge at 80 °C in the basic aqueous environment. TEOS exists as an anionic moiety above the isoelectric point (pH = 2). The fabrication of MSNPs, based on hydrolysis and condensation reactions, was successfully achieved. The removal of surfactant was necessary not only for the generation of porous structure but also due to the toxic effects of CTAB on biological membranes. The MSNPs size was initially determined by dynamic light scattering, where the diameter was 115.70 ± 1.13 nm with a polydispersity index of 0.20 and zeta potential of +29.60 ± 4.30 mV. After the surfactant removal, zeta potential shifted from positive to negative (-21.89 ± 2.32 mV).

The surfactant removal was characterized by FTIR, and the results are shown in Figure 1. CTAB surfactant typically showed two intense peaks in the region of 2800–3200 cm^−1^ corresponding to symmetric (2850 cm^−1^) and asymmetric (2915 cm^−1^) stretching vibrations of CH_2_ chains. The presence of these specific peaks in spectrum of CTAB-MSNPs shows the availability of surfactant template in pores, which was later removed with extraction. In the case of extracted MSNPs, the absence of these CTAB peaks indicates the removal of surfactant (Quan et al., 2015). On the other hand, the FTIR spectrum of CTAB-MSNPs showed that a broad band in the region of 1000–1300 cm^−1^ is a characteristic feature of Si-O-Si. The peaks at 1042 cm^−1^ and 953 cm^−1^ are typically for asymmetric vibrations of Si-O-Si and Si-OH, respectively. The signals at 796 cm^−1^ are attributed to symmetrical stretching of Si-O-Si vibrations, and signals at 440 cm^−1^ are due to bending vibrations of Si-O-Si. Our findings are in accordance with already reported data (Sanaeishoar et al., 2015). FTIR Spectrum is not only showing the surfactant removal but also the formation of silica structure.

Nitrogen adsorption-desorption was performed to evaluate surfactant removal, and surface area characterization and isotherms are shown in Figure 1(b). The results in terms of surface area, volume, pore diameter, and total pore volume are shown in Table 1, where surface area and volume were obtained from BET while pore diameter and total pore volume were obtained from the BJH method. Here we can observe that CTAB-MSNPs without washing exhibited lower surface area (42.75 m^2^/g) because of the available surfactant template in pores with very low volume, but after extraction, this surfactant was removed, and an increase in surface area (1134.43 m^2^/g) was observed. BJH measurement shows that the pore size was about 2–3 nm in diameter, as shown in Figure 1(c).

**Table 1.** Surface area, pore size and volume of MSNPs before and after surfactant removal.

Samples	Surface Area (m^2^/g)	Vm (cm^3^(STP) /g)	Pore diameter d-_peak_ (nm)	Total Pore Vol (cm^3^/g)
**CTAB-MSNPs**	42.75	9.80	2.43	0.34
**MSNPs**	1134.43	260.61	2.43	1.07

**Figure 2.**
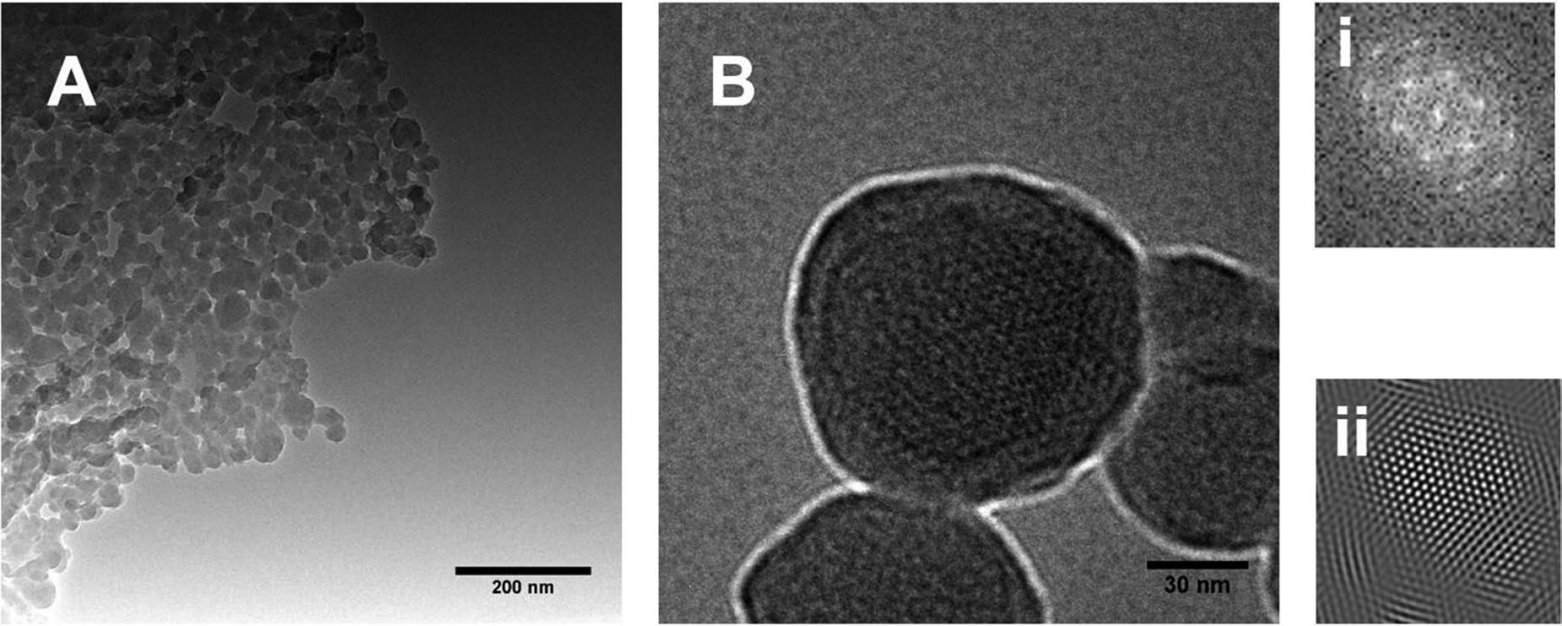
TEM images of (a-b) MSNPs showing the porous structure and (b) (i & ii) are representing FFT and IFFT patterns respectively.

Morphological studies were performed with TEM to visualize the porosity of MSNPs. The porous structure of MSNPs can be observed after surfactant extraction, as shown in Figure 2(a). The pore size was under 5 nm, corresponding to BJH pore size measurement. A uniform porous structure can be observed in Figure 2(b), where insets of Figure 2(b) (i & ii) show Fast Fourier Transform (FFT) and Inverse Fast Fourier Transform (IFFT), respectively. These FFT and IFFT patterns indicate a uniform array of porosity in MSNPs.

### Preparation of Lip_c_

Liposomes as carriers for the CA-IX inhibitor were prepared by the thin-film hydration method, and the same molar mass ratios of DPPC:cholesterol:DOTAP (85:12:3) were used. CA-IX inhibitor as a hydrophobic agent was expected to be loaded in the lipid bilayer. These CA-IX inhibitor liposomes (Lip_c_) were initially characterized by DLS for size distribution and LDV for surface charge. Initially, 2 mM of CA-IX inhibitor liposomes were prepared, and the size was 309.00 ± 24.93 nm, which was higher than we required for MSNPs coating. The PDI for the liposomes mentioned above was 0.54 ± 0.03. Later the size reduction was observed with a lower concentration of the CA-IX inhibitor (20 µM). Here it was noticed that the size of Lip_c_ characterized with DLS was 109.20 ± 9.69 nm with PDI 0.21, which was in the desired range. Although the reason for this size reduction was unclear, however the concentration dependant size and PDI reduction were in accordance with the previous report (Kastner et al., 2015). The surface charge of Lip_c_ was also a critical factor to be characterized for coating negatively charged MSNPs. The surface charge of Lip_c_ observed by LDV was +23.50 ± 7.51 (mV), which was also helpful for the liposomal coating to MSNPs.

### Preparation of Dox-MSNPs and Lip_c_-Dox-MSNPs

For the evaluation of drug entrapment, an indirect method was adopted. As MSNPs are anionic in nature and Dox exists in the form of cations at neutral pH, so Dox can easily be entrapped in the porous structure. Therefore, a shift of the higher negative charge of MSNPs to a lower negative or neutral charge was observed because of Dox entrapment. Here, the drug entrapment was 42.5%, and the loading capacity of MSNPs was 51%, underlying the particles’ higher surface area because of their porous structure (Liu et al., 2016). Previously optimized MSNPs to liposomes molar mass were used for the lipid coating. As the size and surface charge of Lip_c_ were in the same range as of blank liposomes, Lip_c_ can be easily coated onto Dox-MSNPs. According to DLS and LDV results, after Lip_c_ coating, the size of MSNPs was increased to 126.39 ± 8.73 with a PDI of 0.28, and similarly, a relatively high zeta potential of +14.26 ± 3.32 was observed. These results indicate the coating of positively charged liposomes to negatively charged drug-loaded MSNPs. As a fixed lipid to MSNPs ratio was used, therefore the adequate concentrations of both CA-IX inhibitor and Dox were necessary for co-delivery.

### Evaluation of lipid coating

**Figure 3.**
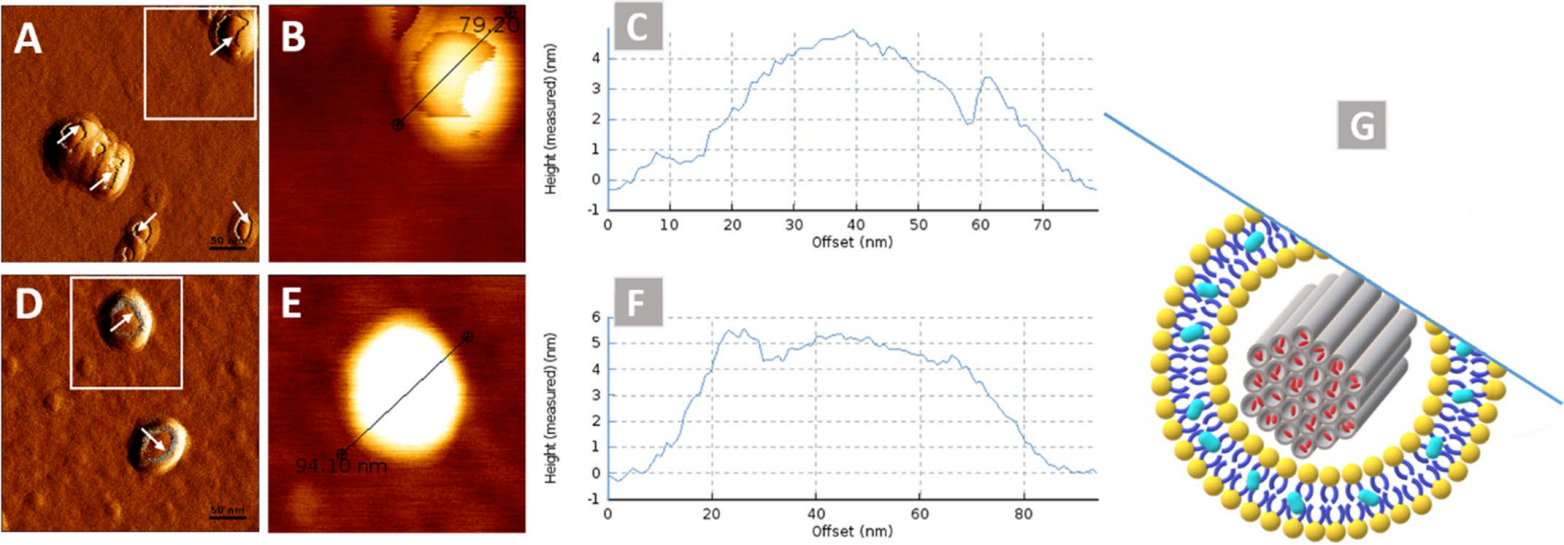
AFM images showing (a & d) amplitude trace, (b & e) height trace, (c & f) respective identified line graph and G as a representative image of surface measurement of lipid coating to MSNPs.

After liposomes preparation and characterizations, the next step was to evaluate the lipid coating to MSNPs. The Lip_c_ coating to MSNPs was visualized by AFM, and micrographs are shown in Figure 3. A uniform layer can be observed over the MSNPs, which indicates that lipid coating onto MSNPs was successfully attained. This technique involves the interaction of the cantilever tip with the sample and causes the damage or deformation of soft structures like liposomes. This feature of AFM was helpful to visualize the lipid coating, as indicated with arrows in Figure 3 (a&d). These images are similar to those of previously reported AFM images where the lipid coated particles had an incomplete lipid layer around them (Baghdan et al., 2018). The lock-In phase image with phase contrast is represented in Figure S2 (Supplemental material) where the difference in contrast is indicating the coating of soft structure (liposomes) over the hard structure (MSNPs).

### *In vitro* drug release

**Figure 4.**
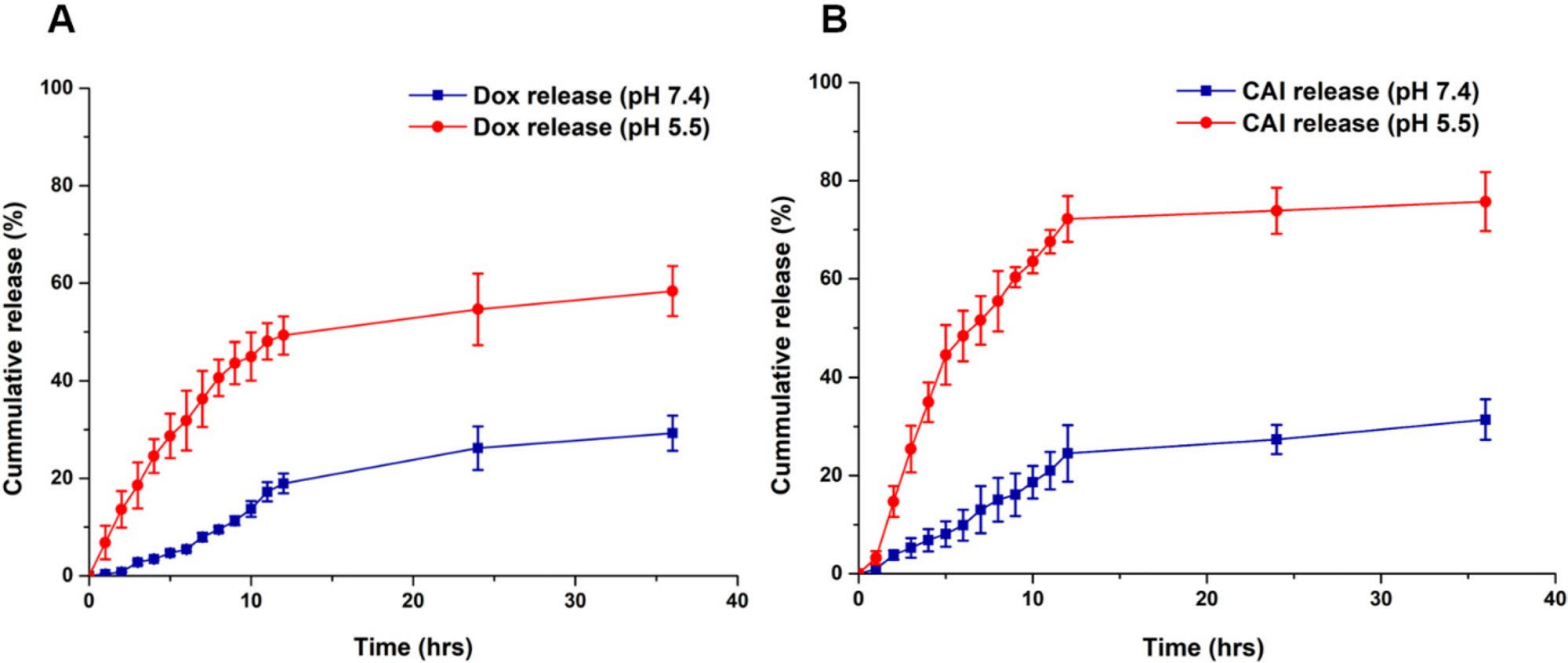
Doxorubicin release profile from Dox-MSNPs and Lip_c_-Dox-MSNPs.

*In vitro* Dox release studies were performed to evaluate the release pattern. The release of Dox from MSNPs was very slow, as shown in Figure S3 (Supplemental material). This sustained release behavior of MSNPs made them suitable candidates for the delivery of chemotherapeutic drugs after reaching the target site and producing anticancer effects for a longer duration. These findings are in accordance with Naz *et al.,* where a sustained release effect of the drug has been reported (Naz et al., 2019). Comparatively, the Dox release rate from Lip_c_-Dox-MSNPs at pH 7.4 was slower than the release from the bared MSNPs as shown in Figure 4(a). This relatively slower release of Dox from Lipc-Dox-MSNPs was due to more retention by lipid coating, which reduced the drug release. Because of its hydrophobic nature, the lipid layer can reduce the permeability of hydrophilic drugs leading to a lesser amount of released drug (Zhang et al., 2014). However, a faster release of Dox was observed at pH 5.5 where almost 50% of the drug was released after 12 h. Similarly, a significant difference in release pattern of CA-IX inhibitor was observed at pH 7.4 and pH 5.5 as shown in Figure 4(b). After 12 h the release at pH 7.4 was about 25% but pH 5.5 enhanced the release up to 72%. These results indicate that the release of Dox and CA-IX inhibitor is pH sensitive (Chang et al., 2022).

## Serum stability studies

As serum proteins play crucial role and can affect the stability of formulations therefore stability studies under physiological conditions were performed. The formulations were incubated with different mediums containing different FCS concentrations. The results of hydrodynamic size, PDI, and zeta potential are shown in Table 2. As the data shows there was not a significant change in the size and PDI of Lip_c_ when incubated in medium with 10% FCS till 24 h however a slight increase in Lip_c_-Dox-MSNPs size was observed. Contrarily, both Lip_c_ and Lip_c_-Dox-MSNPs have shown an increase in size and PDI in medium with 60% FCS specially after 24 h but these effects are relatively lesser in Lip_c_-Dox-MSNPs as compared to Lip_c_. This increase in size can be attributed to protein corona on the formulation surface with higher serum proteins for longer incubation period. On the other hand, a shift to lower zeta potential was observed for both Lip_c_ and Lip_c_-Dox-MSNPs in the medium with 10% and 60% FCS. This reduction in zeta potential is due to deposition of negatively charged proteins to the surface of positively charged nanocarriers which was worse in 60% FCS and 24 h incubation (Ali et al., 2021). As the incubation with 10% FCS for 24 h has not affected the physicochemical characteristics of formulations so they can be subjected to *in vitro* cell culture experiments for mentioned specifications.

**Table 2.** Physicochemical parameter changes of formulations after incubating in PBS, medium with 10% and 60% FCS at 37 °C and 100 rpm for 4 h and 24 h. The hydrodynamic size (intensity distribution), PDI and zeta potential are presented as mean ± SD (*n* = 3).

Medium	Time (h)	Lip_c_			Lip_c_-Dox-MSNPs		
		Avg. Size (nm) ± SD	PDI ± SD	Zeta. Pot (mV) ± SD	Avg. Size (nm) ± SD	PDI ± SD	Zeta. Pot (mV) ± SD
PBS	0	109.2 ± 9.69	0.21 ± 0.01	23.50 ± 7.51	126.39 ± 8.73	0.28 ± .02	14.26 ± 3.32
	4	110.91 ± 4.65	0.23 ± 0.01	20.44 ± 4.26	134.68 ± 12.83	0.26 ± 0.02	3.75 ± 0.61
	24	133.2 ± 2.5	0.26 ± 0.04	5.0 ± 0.57	139.23 ± 2.25	0.30 ± 0.01	4.8 ± 1.42
Medium + 10% FCS	0	109.2 ± 9.69	0.21 ± 0.01	23.50 ± 7.51	126.39 ± 8.73	0.28 ± .02	14.26 ± 3.32
	4	116.95 ± 3.23	0.235 ± 0.01	4.26 ± 0.70	147.36 ± 9.72	0.229 ± 0.06	4.33 ± 0.80
	24	113.97 ± 7.78	0.23 ± 0.01	3.575 ± 1.38	149.23 ± 10.0	0.28 ± 0.016	2.44 ± 1.51
Medium + 60% FCS	0	109.2 ± 9.69	0.21 ± 0.01	23.50 ± 7.51	126.39 ± 8.73	0.28 ± .02	14.26 ± 3.32
	4	185.3 ± 65.74	0.33 ± 0.06	2.58 ± 1.55	121.13 ± 8.7	0.25 ± 0.02	1.98 ± 1.48
	24	220.17 ± 23.35	0.46 ± 0.18	−3.12 ± 7.56	147.93 ± 31.93	0.33 ± .03	0.11 ± 1.44

### Cell culture experiments

#### Western blotting (HIF-1α and CA-IX)

**Figure 5.**
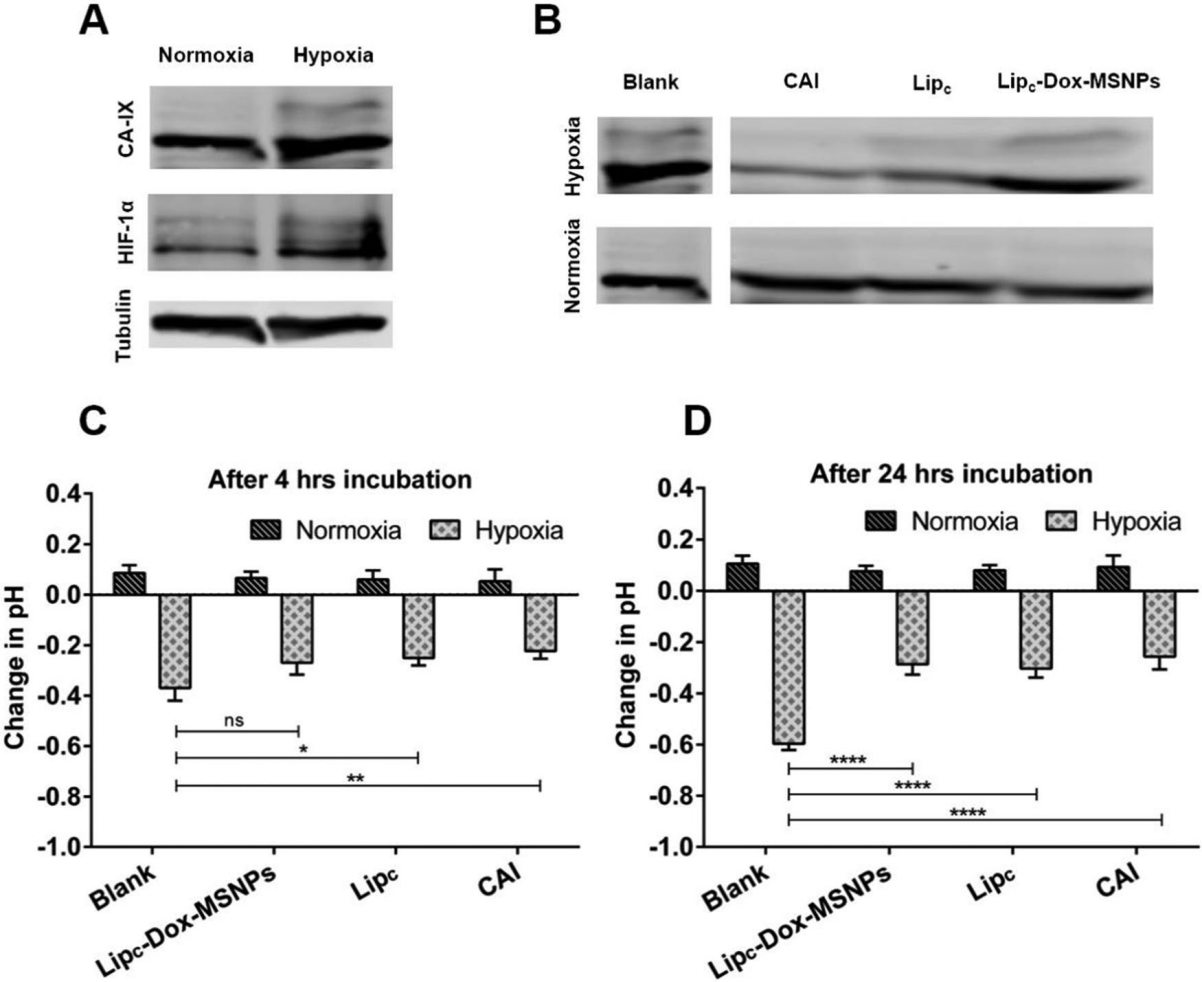
Showing (a) Western blots of CA-IX and HIF-1α expression under normoxia and hypoxia, (b) CA-IX expression after treatment, (c and d) Extracellular acidification in normoxia and hypoxia before and after traetment with CAI, Lip_c_ and Lip_c_-Dox-MSNPs after 4 h and 24 h incubation respectively. Values are represented as mean ± SD (*n* = 3) and statistical significance *p* values (*p* < 0.05) are presented as ***p < 0.001, **p < 0.01, *p < 0.1 and ^ns^p = non-significant.

Protein expression analysis was performed by the western blot technique to validate the overexpression of the CA-IX enzyme. MDA-MB 231 cells are reported to overexpress the CA-IX enzyme under hypoxic conditions (Gieling et al., 2012). For the effectiveness of the CA-IX enzyme inhibitor, it was necessary to confirm the overexpression of the CA-IX enzyme. HIF-1α is another indicator, typically overexpressed in hypoxic conditions (Joshi et al., 2020). The results of western blots for normoxic and hypoxic cells are shown in Figure 5, where both HIF-1α and CA-IX enzymes were overexpressed under hypoxic conditions (Figure 5(a)).

In Figure 5(b), it is noticeable that after 24 h treatment with CA-IX inhibitor, no significant effects were observed under normoxic conditions. However, a significant difference in CA-IX expression was observed in hypoxia between treated and non-treated samples. Cells treated with CA-IX inhibitor, Lip_c_ and Lip_c_-Dox-MSNPs have shown lesser expressions than blank cells, and these results are due to the inhibition of the CA-IX enzyme. In normoxic conditions, treated and non-treated cells have shown no significant difference because the CA-IX enzyme was not overexpressed. Hypoxic conditions mimic the expression of the CA-IX enzyme, which is necessary for the CA-IX inhibitor to perform its inhibitory action (Supuran, 2017).

#### Extracellular acidification test

Hypoxic condition of the cells is directly associated with extracellular pH regulation, which results in reduced efficacy of chemotherapy and radiotherapy. The activity of CA-IX is one of the key factors for an extracellular acidic environment. Cells were treated with different formulations containing CA-IX inhibitor under hypoxic and normoxic conditions (McDonald et al., 2019) for 4 h and 24 h, and the effect of the CA-IX inhibitor on the extracellular acidic pH was evaluated. As results show in Figures 5(c & d), an increase in extracellular pH has been observed by inhibiting the CA-IX enzyme in hypoxia. This effectiveness in pH change is only possible in hypoxic conditions with enzyme overexpression related to oxygen-deprived conditions (Rami et al., 2013). The data indicated that in hypoxia, CA-IX was activated, but the CA-IX inhibitor ultimately reduced the extracellular acidosis and these effects were more significant after 24 h. It was also observed that the change in extracellular pH under normoxic conditions even after treatment with the CA-IX inhibitor was non-significant. This also indicates that the inhibition of the CA-IX enzyme is linked with its overexpression in hypoxic conditions, where it maintains the balance between internal alkaline and external acidic environment.

#### *In vitro* cytotoxicity assay (MTT assay)

**Figure 6.**
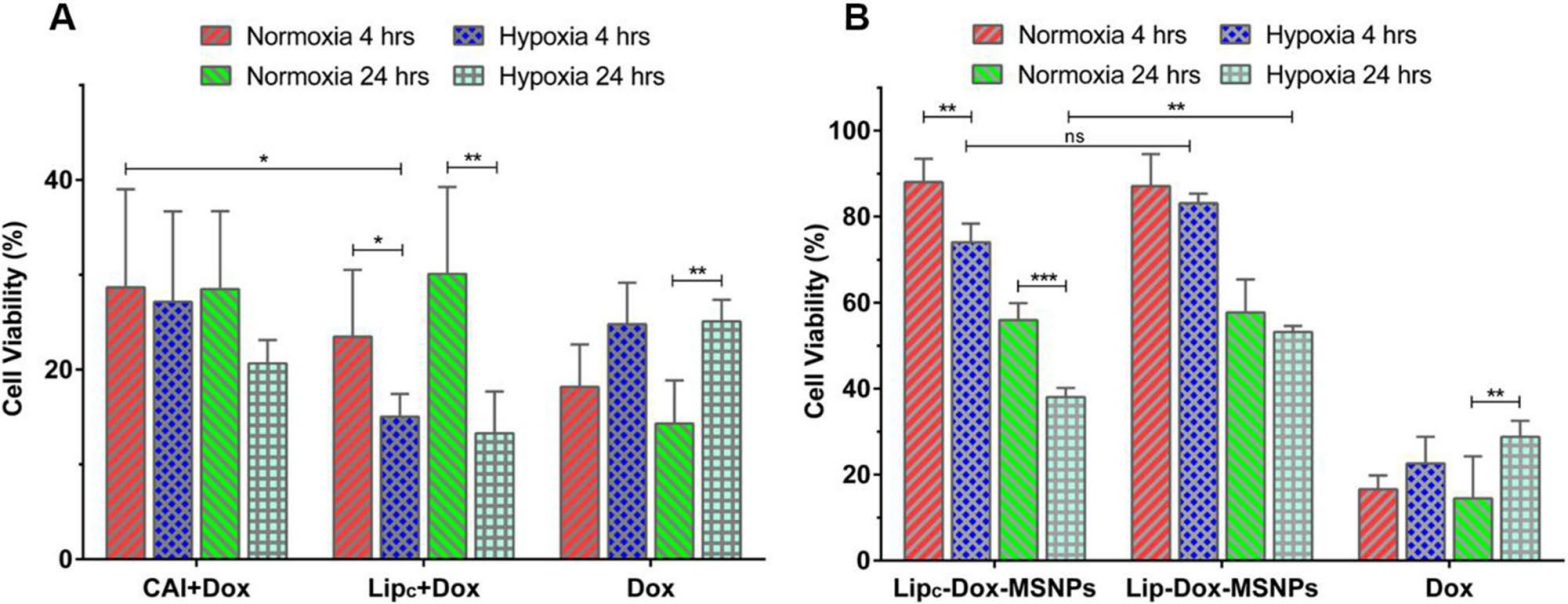
*In vitro* cytotoxicity evaluation of (a) Dox (4 h & 24 h incubation) with CAI and Lip_c_ pretreated cells in normoxia and hypoxia and (b) Lip_c_-Dox-MSNPs, Lip-Dox-MSNPs and Dox after 4 h & 24 h incubation in normoxia and hypoxia. Values are represented as mean ± SD (*n* = 3) and statistical significance *p* values (*p* < 0.05) are presented as ***p < 0.001, **p < 0.01, *p < 0.1 and ^ns^p = non-significant.

Many of the chemotherapeutic agents, specifically designed to target highly proliferating cells, fail in hypoxia even after reaching the cells. The effective concentration of drugs taken up by the tumor cells is a prerequisite for suitable effectiveness (Alfarouk et al., 2015). Before starting the *in vitro* cell culture experiments, the inertness of the nanocarriers was assessed. Briefly, MSNPs, Lip_c_ and Lip_c_-MSNPs were incubated with cells for 4 h and cell viability was evaluated by MTT assay. The results shown in Figure S4 (Supporting Information) indicated that all the nanocarriers even at higher concentrations are nontoxic in nature. Afterwards, the cytotoxic effects of pure Dox with and without free CA-IX inhibitor and Lip_c_ in both normoxia and hypoxia were evaluated by cell viability assay. The cytotoxic effects of Dox after 4 h and 24 h incubation with MDA-MB 231 cells, pretreated with CA-IX inhibitor and Lip_c_ for 4 h in normoxia and hypoxia, are shown in Figure 6(a). The final concentrations of Dox and CA-IX inhibitor were 100 µg/mL and 360 nM respectively. Here it was observed that the pure Dox had shown more cytotoxic effects in normoxia as compared to hypoxia with 4 h incubation period. Similar results were observed with 24 h Dox incubation time. The lower cytotoxic effects of Dox in hypoxia are due to extracellular acidosis as evaluated in the extracellular acidification test. The acidic environment causes ionization of weakly basic drugs like Dox and, therefore, lesser cellular uptake of ionized Dox. Another possibility for lesser Dox toxicity in hypoxia would be the suppression of ROS-mediated cytotoxicity of Dox in hypoxia, as already reported (Khan et al., 2019; Xu et al., 2019; He et al., 2020). These results were different from those we got after inhibition of CA-IX. After treatment with pure CA-IX, Dox with 4 h incubation has shown no significant difference in cytotoxicity between normoxia and hypoxia; however, after 24 h Dox incubation, more cytotoxic effects of Dox have been observed in hypoxia as compared to normoxia.

On the other hand, with pretreated Lip_c_ in comparison to pure CA-IX inhibitor, Dox has produced higher toxic effects after 4 h and 24 h incubation and these results were more significant after 24 h. These findings indicate that in hypoxic conditions, inhibition of the CA-IX enzyme is beneficial for more cytotoxic effects of Dox, and these effects can be further enhanced by loading CA-IX inhibitors to liposomes. The better results with liposomes, as previously reported, are due to higher biocompatibility and internalization because of the structure of the lipid layer (Liu et al., 2020). After establishing this fact, the next step was to evaluate the cytotoxic effects of Lip_c_-Dox-MSNPs in a hypoxic environment.

In this part of studies, as shown in Figure 6(b), a similar pattern has been observed, where pure Dox in hypoxia has shown lesser cytotoxic effects due to ionization and suppression of ROS-mediated toxicity of Dox after 4 h and 24 h incubation. Lip_c_-Dox-MSNPs have shown more cytotoxic effects in hypoxia than normoxia in 4 h and 24 h incubation. On the other hand, Lip-Dox-MSNPs have shown almost the same cytotoxic effects in hypoxia and normoxia after 4 h incubation. 24 h incubation of Lip-Dox-MSNPs has also demonstrated the same pattern in hypoxia and normoxia. A cross-comparison between Lip_c_-Dox-MSNPs and Lip-Dox-MSNPs in normoxia with 4 h and 24 h incubation showed no significant difference, but when we compare Lip_c_-Dox-MSNPs and Lip-Dox-MSNPs in hypoxic conditions, a considerable difference can be observed with more Lip_c_-Dox-MSNPs cytotoxic effects after 24 h. These results also indicate that the CA-IX inhibitor shows its effects only in hypoxia when CA-IX is overexpressed. Our findings are in accordance with previously established results (Janoniene et al., 2017; Shabana et al., 2018) where the effects of Dox in combination with CA-IX inhibition have produced better cytotoxic effects. By these synergistic effects of Lip_c_-Dox-MSNPs, a CA-IX inhibitor- and Dox-loaded drug delivery system is a suitable system for the delivery of weakly basic drug under hypoxic conditions.

#### Cellular uptake studies

**Figure 7.**
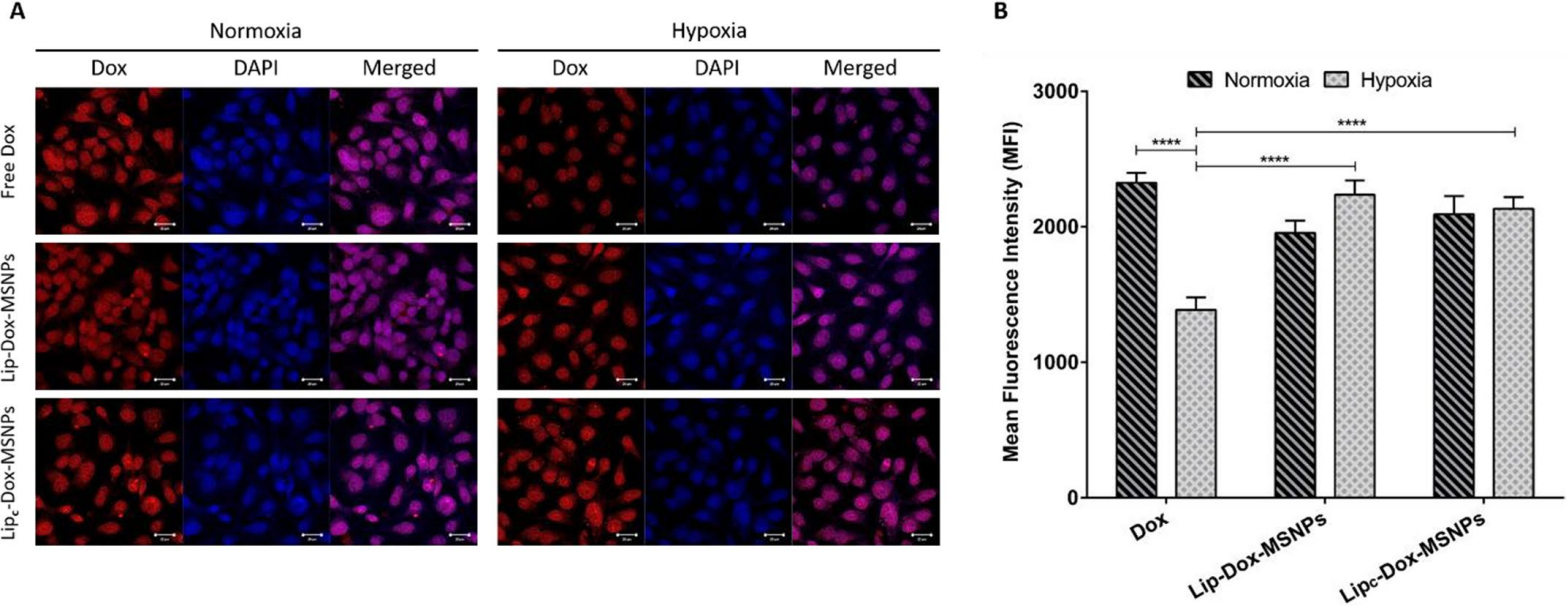
*In vitro* cellular uptake of doxorubicin where (a) is the representing qualitative uptake of Dox with different formulations in normoxia and hypoxia observed witgh CLSM and (b) Quantitaive evaluation of mean fluorescenc intensity (MFI) of Dox uptake with different formulation measured with FACS. Values are represented as mean ± SD (*n* = 3) and statistical significance *p* values (*p* < 0.05) are presented as ****p < 0.0001.

The intracellular uptake of the nanocarriers and Dox release were evaluated by CLSM images and flow cytometry. As shown in Figure 7(a), the Dox fluorescence in the nuclear region in normoxia indicates higher uptake of free Dox. However, in hypoxic conditions, lesser Dox intensity was observed because of lesser uptake of free Dox which is attributed to microenvironment pH changes. As stated in the literature, hypoxic conditions can result in different kinds of drug resistance because of tumor microenvironment pH, which hinders drug uptake. These results are in line with the cytotoxic assay, where lower cellular toxicity of Dox was observed in hypoxia compared to normoxia. In contrast, an increase in Dox intensity with Lip-Dox-MSNPs and Lip**_c_**-Dox-MSNPs was observed in hypoxia. The shielding effect of the lipid layer protected the drug from its exposure to extracellular lower pH and enhanced the cellular uptake. The biocompatible and proto cellular nature of liposomes increases cell membrane interaction and internalization. It was also observed that non-lipid coated MSNPs showed lesser uptake (data not shown). The higher uptake of lipid coated nanoparticles has already been reported (Ali et al., 2021). A similar pattern was observed in mean fluorescence intensity (MFI) based flow cytometric analysis. As shown in Figure 7(b), the intensities of Lip-Dox-MSNPs and Lip**_c_**-Dox-MSNPs in normoxia and hypoxia were higher, which are comparable to free Dox in normoxia; however, free Dox in hypoxia has shown lesser intensity. These quantitative results and histograms (Figure S5 in Supplemental material) also indicate the effectiveness of nanocarriers in hypoxia. Although free Dox in normoxia and hypoxia has produced more toxicity than lipid coated carriers, but higher intensities with nanocarriers show higher uptake where the drug would be released in a sustained manner in the intracellular region. Similar findings were observed in our other experiments, where lipid coating resulted in higher cellular uptake, but higher toxic effects were observed with a longer incubation period (Amin et al., 2021). Furthermore, under hypoxia, Lip-Dox-MSNPs have shown almost similar MFI compared to Lip**_c_**-Dox-MSNPs, which is contrary to cytotoxic results. The lesser cytotoxic effects of Lip-Dox-MSNPs are due to the suppression of ROS-mediated effects in oxygen-deprived environments. The co-delivery of CA-IX enzyme inhibitor and Dox enhanced the cytotoxicity by reviving ROS-mediated Dox effects.

### Hemocompatibility assay

**Figure 8.**
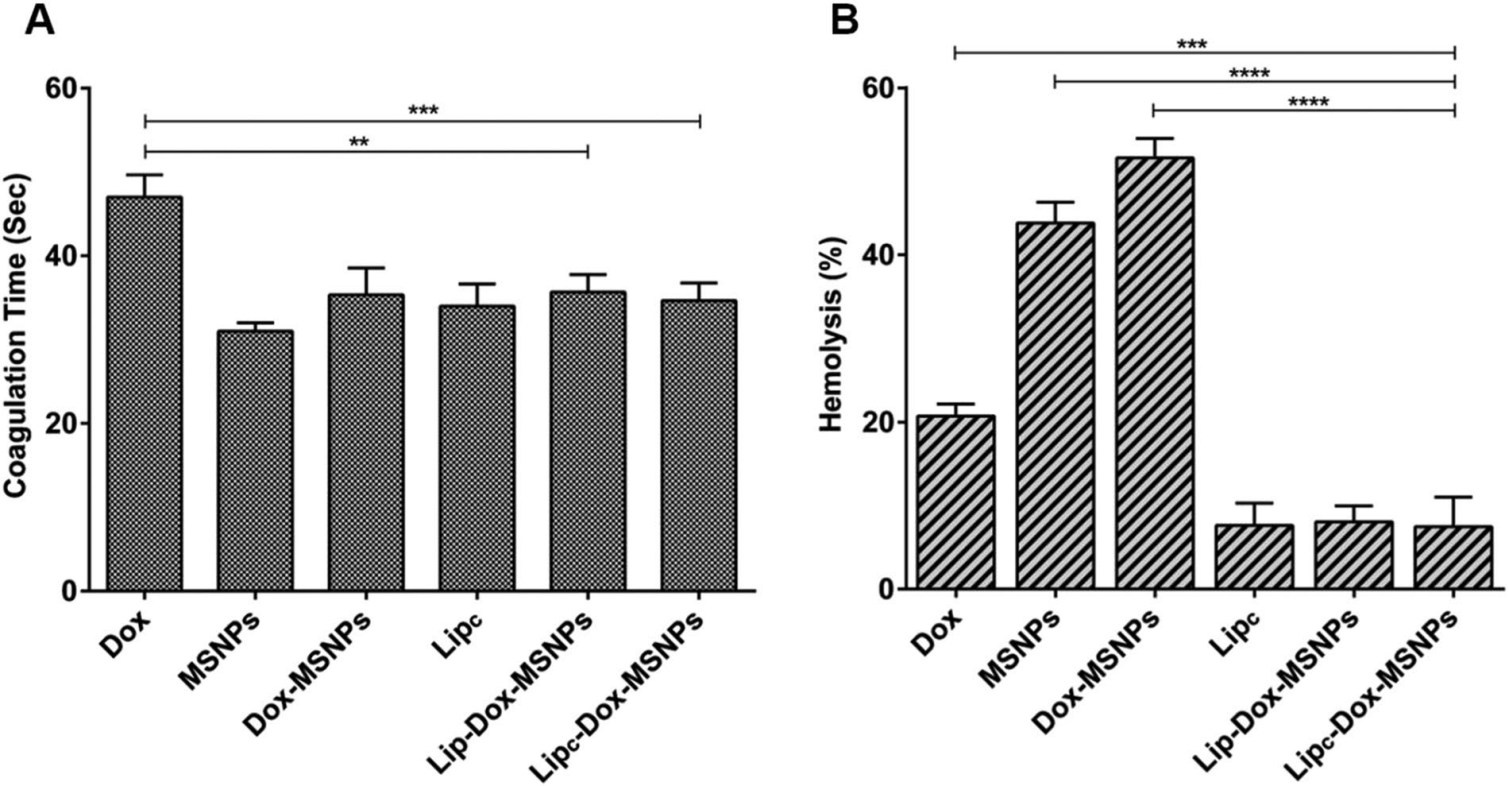
Hemocmpatibility assay showing (a) coagulation time and (b) hemolysis effects of different formulations and free Dox. Tritox-X100 and blood plasma were considered as negative and positive control. All the values are represented as mean of triplicate along with standard deviation. Values are represented as mean ± SD (*n* = 3) and statistical significance *p* values (*p* < 0.05) are presented as ****p < 0.0001, ***p < 0.001, **p < 0.01.

The effects of formulations on the coagulation time were evaluated by aPTT assay. As shown in Figure 8(a), all the formulations have shown coagulation time between 30 sec to 40 sec, while the coagulation time of free Dox was about 48 sec. The aPTT values above 50 sec are clinically significant, but values above 70 sec result in hemorrhage and continuous bleeding (Ali et al., 2020). The drug loading to MSNPs was helpful to significantly reduce the aPTT values of free Dox, which indicates the suitability of the nanostructures. The results of the hemocompatibility assay show that the lipid coating to MSNPs provided a dual surface for drug loading, enhanced biocompatibility, and minimized the hemolytic effects of free drug and MSNPs.

The determination of hemolysis was based on the extent of erythrocyte destruction and release of hemoglobin because of their interaction with different formulations. Free hemoglobin in the presence of atmospheric oxygen is converted to oxyhemoglobin which can be detected spectrophotometrically. The results of the hemolysis assay are shown in Figure 8(b), where free Dox, MSNPs, and Dox-MSNPs have shown hemolytic effects. The destruction of erythrocytes is attributed to the surface density of the silanol group of MSNPs (Bharti et al., 2015). On the other hand, CA-IX enzyme inhibitor-loaded liposomes have shown lower hemolytic effects. In the lipid coated formulations, the hemolytic effects of free Dox and MSNPs were significantly masked because of the biocompatible nature of liposomes.

## Conclusion

In this study, we have developed a combined drug delivery system to encounter the hypoxia-related resistance of cancer cells to weakly basic anticancer drugs. The co-delivery of the CA-IX inhibitor and Dox was successfully attained by coating carbonic anhydrase inhibitor-loaded liposomes to Dox-loaded MSNPs. *In vitro* cytotoxic experiments were performed under normoxia and hypoxia, and it was shown that the CA-IX enzyme was overexpressed under hypoxic conditions. The enzyme inhibitor can be effective only when the enzyme is overexpressed in hypoxia. Furthermore, the improved inhibitory effects of the CA-IX inhibitor were observed when loaded in liposomes compared to pure enzyme inhibitors, indicating higher cellular uptake because of liposomal biocompatibility. Lesser cytotoxic effects of Dox under hypoxia in the absence of the CA-IX inhibitor are due to acidification of the extracellular environment, resulting in ionization of Dox and ultimately lesser uptake. However, the combined effects of enzyme inhibitor and Dox have synergistic cytotoxic effects under hypoxia because of CA-IX enzyme inhibition.

## Supplementary Material

Supplemental Material

## Data Availability

The data that support the findings of this study are available from the corresponding author, U.B., upon reasonable request.
