# Supplementary material for: Co-delivery of carbonic anhydrase IX inhibitor and doxorubicin as a promising approach to address hypoxia-induced chemoresistance
Source: Drug Deliv. 2022 Jul 17;29(1):2072–85. doi: 10.1080/10717544.2022.2092234 (PMC9297722; doi:10.1080/10717544.2022.2092234)
Supplement: Supplemental Material [file IDRD_A_2092234_SM3145.docx]

**Co-delivery of Carbonic Anhydrase IX Inhibitor and Doxorubicin as a Promising Approach to Address Hypoxia-Induced Chemoresistance**

*Muhammad Umair Amin^a^, Sajid Ali^a,b^, Muhammad Yasir Ali^a,c^, Dominik C. Fuhrmann^d^, Imran Tariq^a,e^, Benjamin S. Seitz^a^, Eduard Preis^a^, Jana Brüßler**^a^, Bernhard Brüne^d^, Udo Bakowsky^a^*

**^a^**Department of Pharmaceutics and Biopharmaceutics, University of Marburg, Marburg, Germany

**^b^**Department of Chemistry, Angström Laboratory, Uppsala University, Uppsala, Sweden

**^c^**Faculty of Pharmaceutical Sciences, GC University Faisalabad, Faisalabad, Pakistan

**^d^**Institute of Biochemistry I, Faculty of Medicine, Goethe-University Frankfurt, Frankfurt, Germany

**^e^**Punjab University College of Pharmacy, University of Punjab, Lahore, Pakistan

**CA-IX Enzyme Inhibitor**

The Carbonic anhydrase IX enzyme inhibitor utilized in this study is a benzene sulfonamide. It is selective for human hCA IX and hCA XII (Ki = 0.9 nM and 5.7 nM, respectively). CA inhibitor AAZ (Ki = 250 nM, 12 nM, 25 nM, and 5.7 nM for hCA I, hCA II, hCA IX, and hCA XII, respectively).

It is highly soluble in organic solvent like DMSO where its solubility is 50 mg/ml. Although its solubility profile in the aqueous medium is not provided however it is recommended that DMSO solution can be diluted in the aqueous medium. Furthermore, the solubility experiments were performed with different solvents including PBS (pH 7.4) and PBS (pH 7.4) containing 0.1% Tween-80 where it was solubilized up to 2.5 mg/ml with 5 mg/ml respectively.


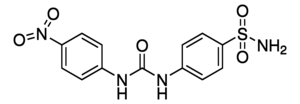


***Figure S1:*** *Structure of CA-IX enzyme inhibitor.*


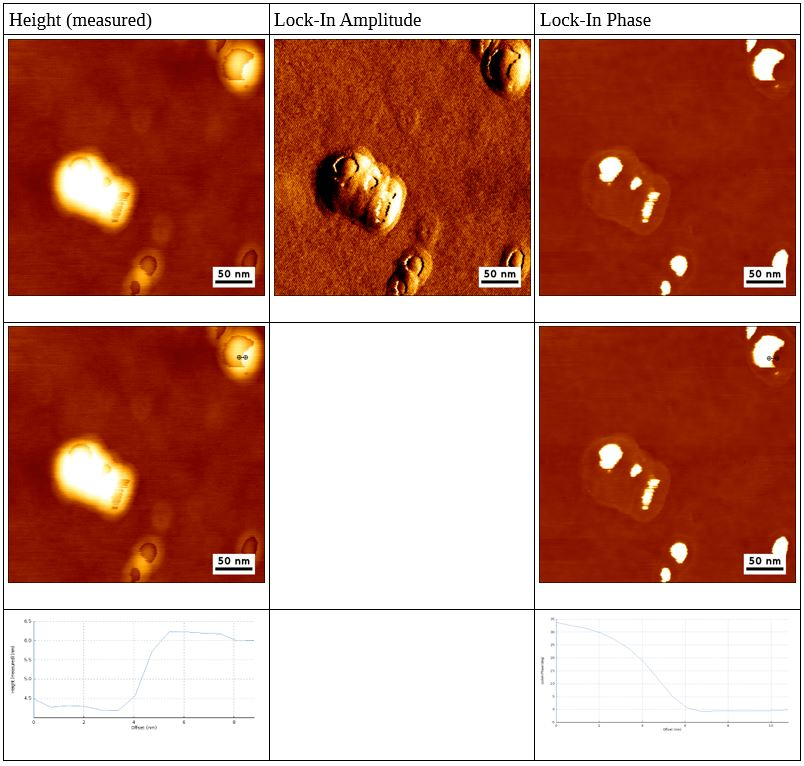


**Figure S2:** *AFM of lipid coated MSNPs including height (measured), Lock-In amplitude and Lock-In Phase images along with graphs. The different bright contrast in Lock-In Phase image is representing soft coating of liposomes over the solid MSNPs*





**Figure S3:** *Doxorubicin release profile from Dox-MSNPs and Lip_c_-Dox-MSNPs* *at pH 7.4*





**Figure S4: *Biocompatibility of nanoformulations (MSNPs, Lip_c_ and Lip_c_-Dox-MSNPs) evaluated by % cell viability with MTT assay. Different concentrations of formulation were used with 4 hrs incubation.***

**Fluorescence Based Cellular Uptake by Flow Cytometry**

Free Dox, Lip-Dox-MSNPs and Lip_c_-Dox-MSNPs were evaluated for cellular uptake of Dox by Mean Fluorescence Intensity (MFI) with flow cytometry. The concentration of Dox used I nthis experiment was 50 µg/ml and cells were incubated for 4 hr in hypoxia and normoxia. Non-treated cells were taken as blank and histograms are presented in Figure 1 where the shift in cell cycle is also indicating the effects of Dox with different formulations.


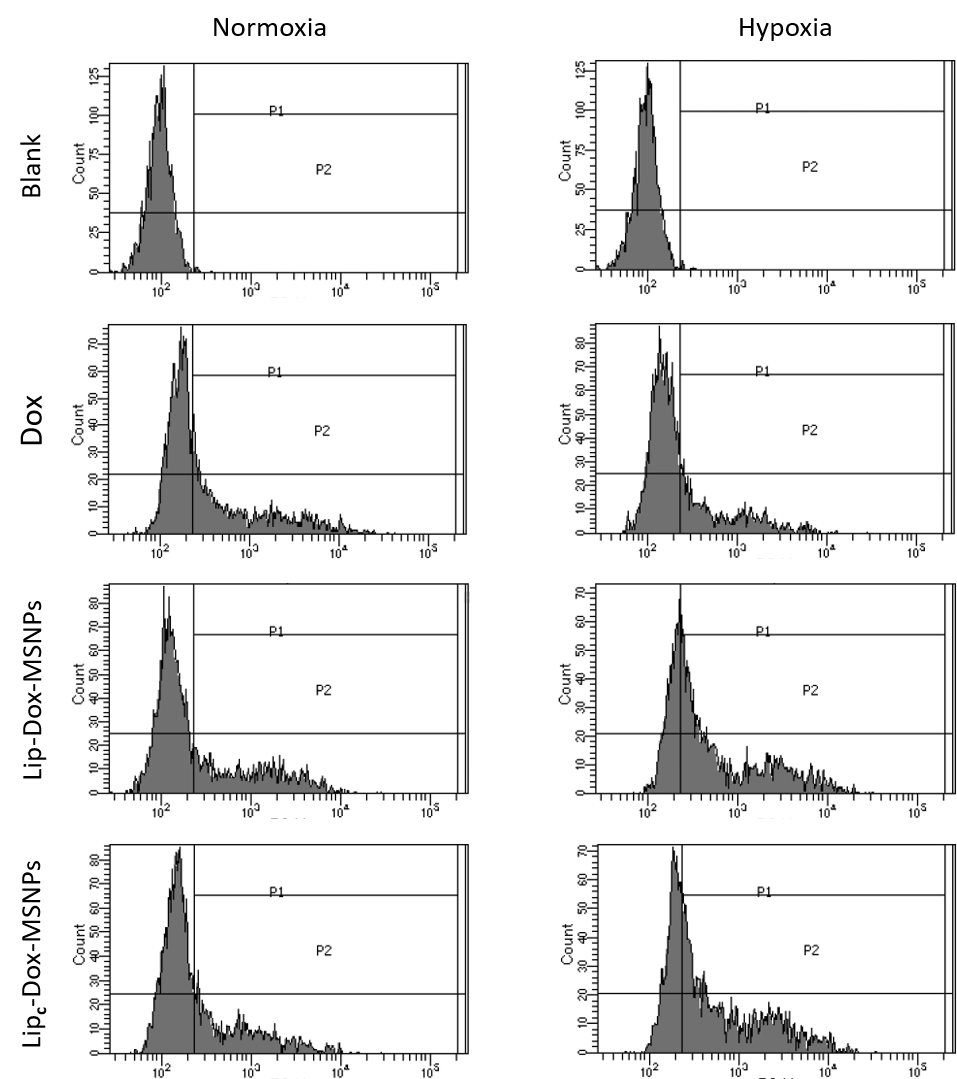


**Figure S5**: Showing cell cycle histograms of cells treated with free Dox, Lip-Dox-MSNPs and Lip_c_-Dox-MSNPs under hypoxic and normoxic conditions. Cells with medium were taken as blank.
